# Ethnic-Based Assessment of Vitamin D and Magnesium Status in the Kingdom of Bahrain

**DOI:** 10.7759/cureus.55967

**Published:** 2024-03-11

**Authors:** Tarik AlShaibani, Rima Abdul Razzaq, Ameera Radhi, Hussain Meer, Abdulaziz Aljawder, Ahmed Jaradat, Yahya M Naguib

**Affiliations:** 1 Physiology Department, Arabian Gulf University, Manama, BHR; 2 Pathology Department, Salmaniya Medical Complex, Manama, BHR; 3 Radiology Department, Alhakeem Radiology Center, Manama, BHR; 4 Family and Community Medicine Department, Arabian Gulf University, Manama, BHR; 5 Clinical Physiology Department, Faculty of Medicine, Menoufia University, Shibin El Kom, EGY

**Keywords:** arabian gulf, bahrain, ethnic-based evaluation, sunlight, magnesium, vitamin d deficiency

## Abstract

Background: Vitamin D deficiency is a major global health problem. Most previous studies focused attention on the significant role of sunlight exposure in the homeostasis of vitamin D and calcium blood levels. Magnesium is pivotal in the proper functioning of vitamin D, and the physiologic functions of different organs require a balanced vitamin D and magnesium status. The relationship between sunlight exposure and blood levels of vitamin D and magnesium has often been overlooked. The aim of this study was to evaluate vitamin D and magnesium status based on sunlight exposure and ethnicity in Bahraini and expatriate workers.

Methods: A cross-sectional study was conducted between October 2018 and September 2019. One hundred and seventy-four subjects participated in this study were subdivided based on their ethnicity and work environment-dependent exposure to sunlight into four groups: (1) Bahraini exposed (n=94), (2) Bahraini non-exposed (n=25), (3) expatriate exposed (n=31), and (4) expatriate non-exposed (n=24). Blood levels of vitamin D and magnesium were evaluated for all the participants.

Results: Independent of ethnicity, vitamin D levels were insignificantly different among the studied groups and were all below the normal reference range. Yet, there was still a sunlight-dependent increase in vitamin D level that could be seen only in Bahraini workers. Magnesium levels were significantly higher in expatriates when compared to Bahraini workers. Sunlight-exposed expatriates had significantly higher magnesium levels than their Bahraini counterparts, while there was no significant difference between both ethnicities in the non-exposed groups.

Conclusion: Country- and ethnic-specific definitions for vitamin D status and sunlight exposure are recommended. The assessment of magnesium status is pivotal in the overall assessment of vitamin D status.

## Introduction

Vitamin D deficiency is a major health problem, and some even argue that it should be recognized as a pandemic [1]. Moreover, the worldwide prevalence of subclinical vitamin D deficiency may reach up to one billion, including both developed and developing countries, increasing the risk of rickets, osteopenia, osteoporosis, and falls and fragility fractures [2,3]. With the advent of the COVID-19 pandemic, a host of studies have investigated the relationship between vitamin D and the severity of COVID-19. A recent study added to the evidence that COVID-19 severity may have ties to vitamin D deficiency [4]. Kumar et al. concluded that people with insufficient levels of vitamin D were more likely to develop COVID-19 than those with high vitamin D intake and that vitamin D improves the immune response against COVID-19 [5]. Furthermore, the immunomodulatory effects of vitamin D have been demonstrated to be advantageous, and low levels of vitamin D could potentially contribute to the poor prognosis of COVID-19 [6]. Although the effects of low vitamin D can be mitigated via supplementation, the role of vitamin D as a therapeutic option in COVID-19 remains controversial [7,8].

Although well known as a vitamin, vitamin D is likely to be one of the oldest hormones ever known [1]. The extent of the literature supporting this suggestion was due to certain characteristics. Firstly, vitamin D is not an essential dietary factor; rather, it is a prohormone produced photochemically in the skin from 7-dehydrocholesterol [1]. Secondly, vitamins are small but indispensable nutrients that organisms cannot produce by themselves, whereas hormones serve as chemical messengers secreted from one part of an organism, usually a gland or certain cells, and circulate in the blood to reach its target tissue and cause the desired response [9]. Thirdly, vitamin D is chemically and structurally related and very similar to the structure of cortisol or other steroid hormones such as testosterone, estrogen, and progesterone [10,11].

Beyond all of this, vitamin D plays important biological roles in bone metabolism, inflammation, and immune modulation. Recent epidemiological studies have demonstrated the relationship between vitamin D deficiency and multiple pathologies [1,12-14].

The synthesis of vitamin D from exposure to sunlight and dietary sources requires magnesium as a cofactor; hence, its deficiency shuts down vitamin D synthesis pathways [15,16]. The activation of vitamin D by magnesium, in turn, helps in calcium and phosphate homeostasis and thereby influences bone formation. Interestingly, all the enzymes that metabolize vitamin D seem to require magnesium [17,18]. It is then essential to ensure that the recommended amount of magnesium is consumed to achieve optimal benefits of vitamin D, albeit deficiencies of magnesium are quite rare because diets usually contain sufficient amount of it [19]. The normal serum concentration of magnesium is 1.7-2.4 mg/dl (0.7-1.0 mmol/L) [20]. Because of its tight homeostatic control, circulating levels of magnesium remain within the normal range, even when the skeletal or intracellular magnesium content of soft tissue is depleted [21].

Vitamin D and magnesium are both needed for the proper functioning of each other, with their adequate balance being essential for maintaining the physiologic functions of various organs. However, studies on the relationship between their levels have produced conflicting reports. A significant association between serum magnesium and vitamin D levels was reported previously [18]. Dai et al. concluded that the right amount of magnesium may be needed to optimize vitamin D levels [22]. Similarly, previous reports suggested that vitamin D is very potent at increasing magnesium absorption [23-25]. On the contrary, Wilz et al. concluded that there was no significant correlation between magnesium absorption and plasma vitamin D concentration [26].

Herein, we tested the hypothesis that differences in exposure to sunlight status/duration may alter vitamin D and magnesium homeostasis. Additionally, we tested the possibility that ethnicity could affect vitamin D levels either directly through differences in skin pigmentation or indirectly through differences in magnesium levels.

## Materials and methods

Ethical considerations

This study received ethical approval from the Research and Ethics Committee at the College of Medicine and Medical Sciences, Arabian Gulf University, Kingdom of Bahrain (E017-PI-11/16). All the procedures were in accordance with the Declaration of Helsinki. Informed consents were obtained from every participant, and each participant was fully informed about all aspects of the study and granted the right to be enrolled or to quit. As an incentive, all participants received a copy of their laboratory test results.

Study design and setting

This study was conducted at the Physiology Department, College of Medicine and Medical Sciences, Arabian Gulf University and the Middle East Hospital, Kingdom of Bahrain. It was an observational cross-sectional study conducted from October 1, 2018 to September 30, 2019 (12-month duration). Randomly selected Bahraini and expatriate male workers from both local companies and the Ministry of Work, Municipality Affairs, and Urban Planning in the Kingdom of Bahrain were enrolled in this study. Participating subjects were further subdivided according to their work environment into exposed to sunlight or non-exposed.

Inclusion criteria

Bahraini and expatriate male workers attending the Middle East Hospital, Kingdom of Bahrain, between October 1, 2018 and September 30, 2019 were included in this study.

Exclusion criteria

The exclusion criteria were subjects aged >60 years, with hyperparathyroidism, or on vitamin D supplementation prior to the study.

Study groups

The sample size was estimated as described previously [27]. A total of 147 workers were randomly selected for the present study. According to the work environment-dependent exposure to sunlight (8-hour direct exposure) and ethnicity, the participants were divided into four study groups: Bahraini exposed (n=94), Bahraini non-exposed (n=25), Expatriate exposed (n=31), and Expatriate non-exposed (n=24).

Blood sampling and biochemical assays

Blood samples were withdrawn from the antecubital vein by standard venipuncture. Serum was obtained by centrifugation of blood samples (3000 rpm for 10 minutes). Samples were immediately frozen at -20°C for subsequent testing. Vitamin D level was measured using the ADVIA Centaur Vitamin D assay (Siemens Healthcare SA, Zurich, Switzerland). The serum concentration of magnesium was determined using a Magnesium Assay Kit (Abcam, Cambridge, United Kingdom), according to the manufacturer's instructions.

Statistical analysis

Data were analyzed using IBM SPSS Statistics for Windows, Version 23.0 (Released 2015; IBM Corp., Armonk, New York, United States). Quantitative variables were presented as mean±SD. A two-way analysis of variance (ANOVA) was conducted on the influence of the two independent variables (ethnicity and sun-exposure status) on each of the levels of vitamin D and magnesium separately in workers in Bahrain. The two levels for ethnicity variable were (Bahraini and expatriate) and the two levels for Sun-exposure variable were (exposed and non-exposed). A P-value ≤0.05 was considered statistically significant. 

## Results

Effects of ethnicity and sun exposure

**Figure 1:**
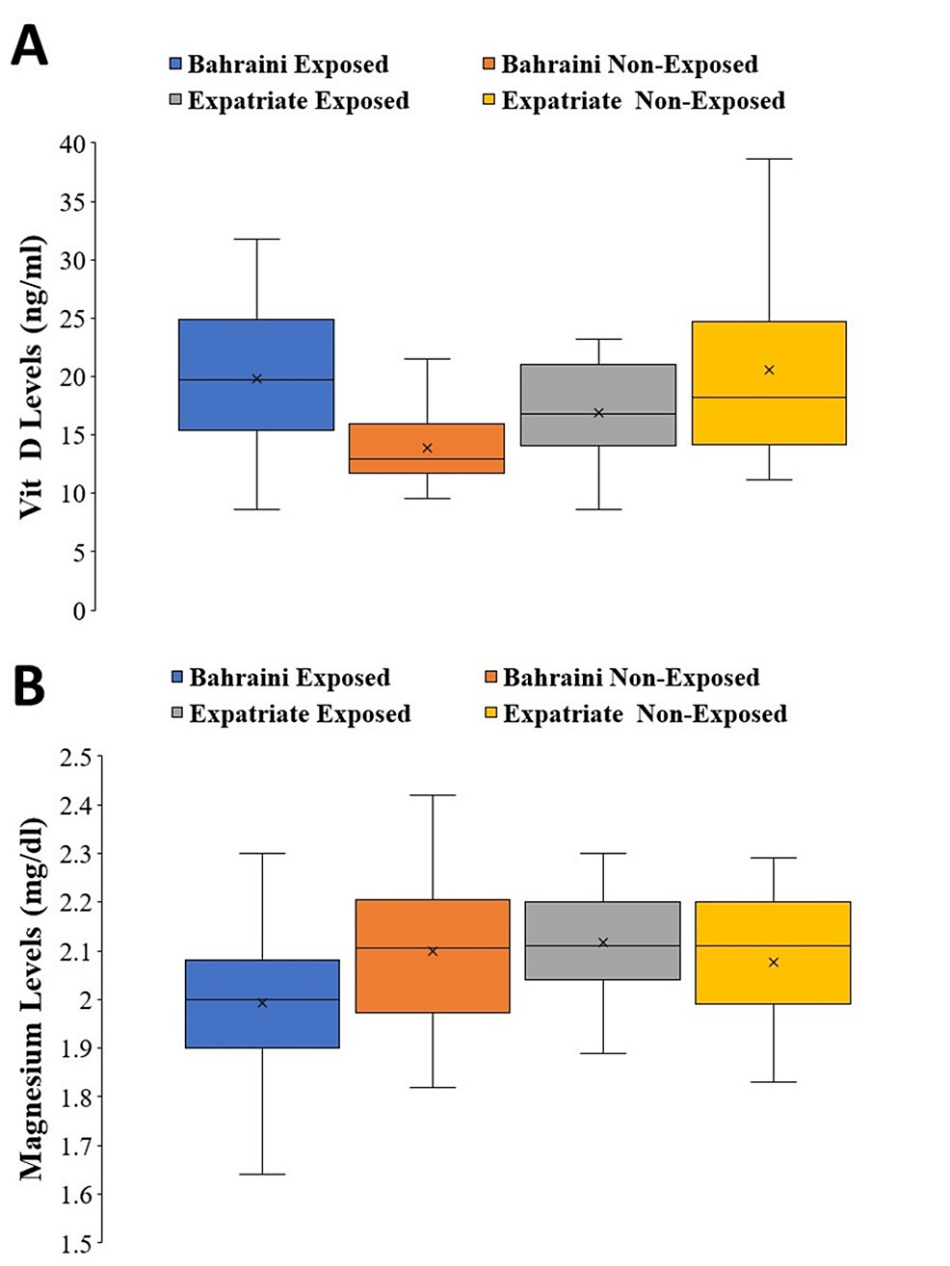
Box and whiskers plot for (A) vitamin D and (B) magnesium levels. “x” represents the mean of the data. Bahraini exposed (n=94), Bahraini non-exposed (n=25), expatriate exposed (n=31), and expatriate non-exposed (n=24).

Figure 1 shows the levels of vitamin D and magnesium in a group of workers in Bahrain, according to sun-exposure status and ethnicity. The two-way ANOVA analysis reveals no main effect of ethnicity on vitamin D levels (F(1, 168)=2.65, P=0.11, partial η^2^=0.016), indicating that jointly Bahrainis (18.64 ± 6.11; n=118) and expatriates (18.49±6.60; n=54) did not differ in their vitamin D levels. Similarly, there was no main effect of sun-exposure status on vitamin D levels (F(1, 168)=0.74, P=0.39, partial η^2^=0.004), indicating that sun-exposed (19.08 ± 5.00; n = 124) and non-exposed subjects (17.32±7.19; n=48) did not jointly differ in vitamin D levels (Figure 1A). For magnesium levels, the ANOVA analysis shows a significant main effect of ethnicity (F(1, 168)=4.93, P=0.03, partial η^2^=0.03), with magnesium levels being lower in Bahraini workers (2.01±0.15; n=118) in comparison with expatriate workers (2.10±0.12; n=54). There was a non-significant main effect of sun-exposure status (F(1, 168)=1.48, P=0.23, partial η^2^=0.01) on magnesium levels (Figure 1B).

**Figure 2:**
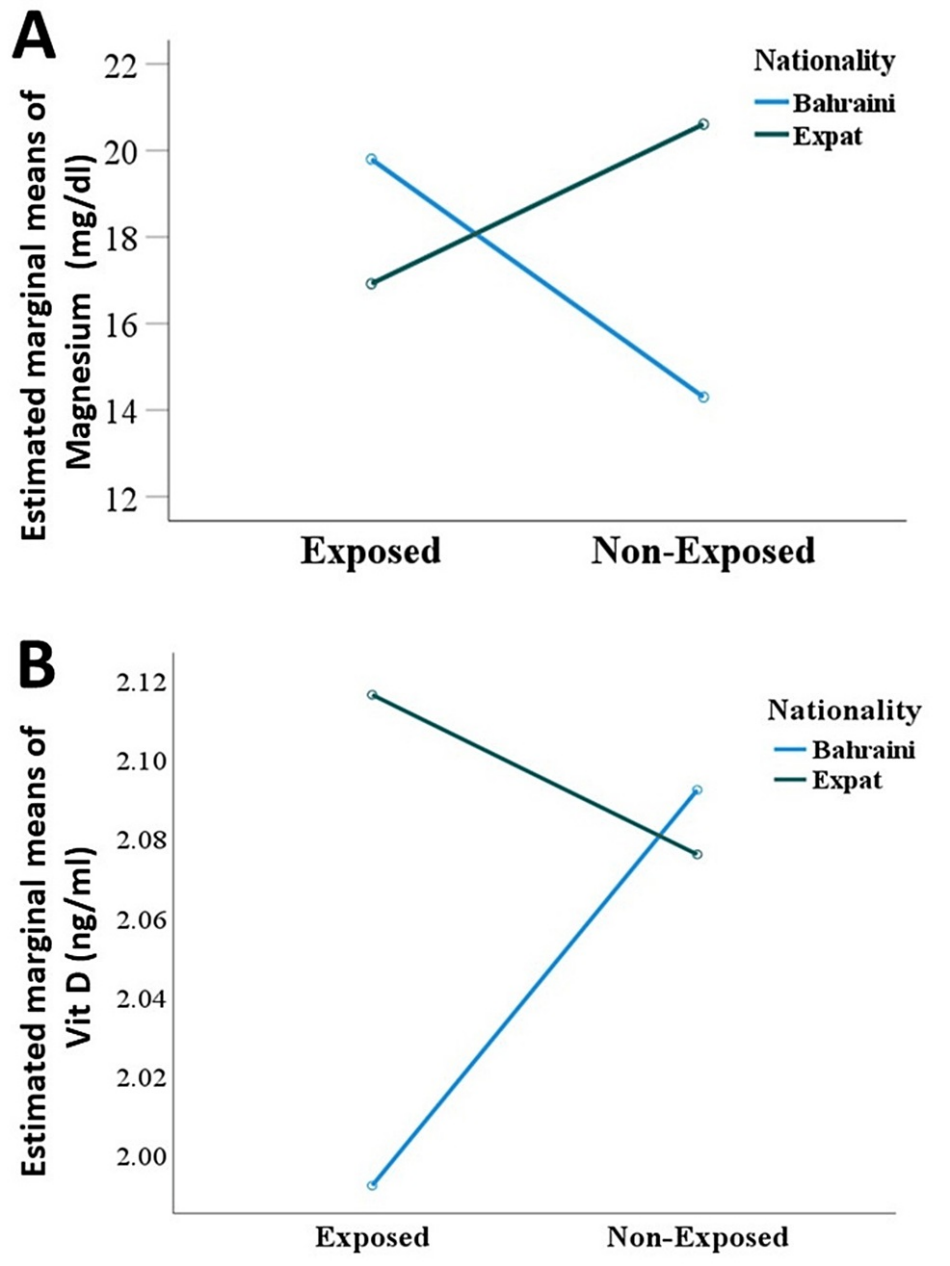
Interaction plot of estimated marginal means of (A) vitamin D and (B) magnesium levels calculated for ethnicity at each status of sun exposure.

However, as illustrated in Figure 2A, there was a significant interaction between ethnicity and sun-exposure status on the levels of vitamin D (F(1, 168)=19.02, P<0.001, partial η^2^=0.10). Tukey-Kramer post hoc analysis showed that vitamin D levels were significantly higher (P<0.001) in sun-exposed Bahrainis than non-exposed Bahrainis by 5.88 ng/ml. However, exposed expatriates had a vitamin D level lower by 3.69 ng/ml than non-exposed expatriates, and this difference was not statistically significant (P>0.05). Exposed Bahrainis and expatriates did not significantly differ in their vitamin D level (P>0.05), but non-exposed Bahrainis and expatriates differed significantly (P<0.001) with a mean difference of 6.68 ng/ml.

As shown in Figure 2B, there was a significant interaction (F(1, 168)=8.19, P=0.005, partial η^2^=0.05) between ethnicity and sun exposure on the levels of magnesium. Post hoc analysis showed that sun-exposed expatriate workers had magnesium levels significantly higher (P<0.001) by 0.12 mg/dl compared to exposed Bahraini workers, but there was no significant difference between the non-exposed Bahraini and non-exposed expatriate workers (P>0.05). Sun-exposed Bahraini workers had significantly higher levels of magnesium (P<0.01) than non-exposed Bahraini workers; however, there was no significant difference in magnesium levels between sun-exposed and non-exposed expatriate workers (P>0.05).

Relationship between magnesium and vitamin D levels

**Figure 3:**
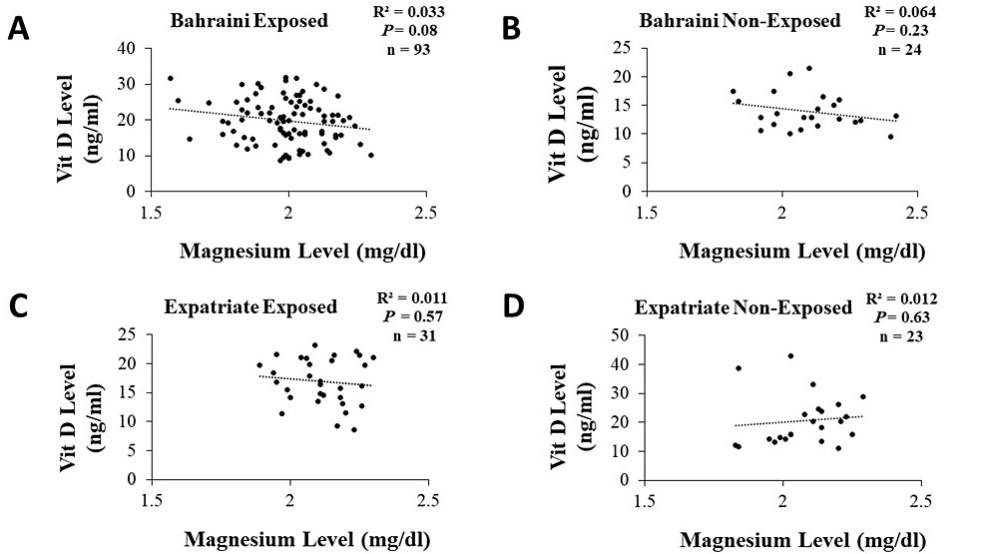
Relationship between vitamin D and magnesium levels in (A) exposed Bahraini, (B) non-exposed Bahraini, (C) exposed expatriate, and (D) non-exposed expatriate workers in Bahrain and different status of sun exposure.

Figure 3 illustrates the correlation between magnesium and vitamin D levels in each of the four groups of workers. In all cases, there was no significant correlation between the two variables.

## Discussion

Vitamin D deficiency is a matter of concern not only in the Arabian Gulf region but also globally. The relationship between the effect of exposure to light on vitamin D and calcium levels has been profoundly studied, and most previous studies have shown that the duration of exposure to the sun causes a significant increase in vitamin D level in the blood [28,29]. Nevertheless, this sun-induced vitamin D synthesis could be greatly affected by the season, time of day, skin color, use of sunscreen, latitude and altitude, air pollution, glass and plastic barriers, and aging [9]. Unfortunately, few studies have shed light on the relationship between exposure to sunlight and its effect on both vitamin D and magnesium levels. In spite of the exposure to ample sunlight in the area, vitamin D deficiency can be considered as an endemic in Saudi Arabia, and vitamin D supplementation has been recommended, particularly for those with diabetes mellitus and hypercholesterolemia [30]. Interestingly, ethnic differences in magnesium intake have been reported in older adults in the USA, where magnesium intake was lower among African American older adults and higher among Hispanics and those from other ethnic groups, compared with Caucasian older adults [31]. Most expatriate workers/employees in Bahrain are of different ethnicities. Just like Bahraini employees, expatriates may work indoors or outdoors, and hence their exposure to the sunlight is also inconsistent.

Vitamin D is a fat-soluble vitamin/hormone and is largely considered as a bone mineralizing, anti-inflammatory, and immune modulatory molecule [12]. Vitamin D deficiency has been linked to increased cardiovascular mortality, cancer, and autoimmune disorders such as multiple sclerosis [32]. The long-held assumption that vitamin D deficiency is not a widespread problem in sunny countries has led to inadequate testing of vitamin D concentration in those populations [33]. Indeed, it may be inappropriate to assume that exposure to sunlight is the most important determinant of vitamin D status since several factors besides sunlight exposure may affect vitamin D production [34]. Herein, we demonstrated country- and ethnic-specific evaluation of vitamin D and magnesium serum levels and investigated the possible correlation between exposure to sunlight, ethnicity, and vitamin D and magnesium status.

Interestingly, and irrespective of ethnicity, there was an insignificant difference in the serum levels of vitamin D between sun-exposed and non-exposed subjects. And although vitamin D levels in the groups exposed to sunlight tended to be higher, vitamin D levels in all the groups were still below the acceptable normal ranges. Most of the beneficial effects of vitamin D are evident when blood levels of vitamin D are at about 30 ng/ml [3]. Vitamin D levels below 30 ng/ml are considered insufficient, while those below 20 ng/ml are considered deficient, and those below 7 ng/ml are considered as severely deficient [35]. The US Institute of Medicine developed a dietary reference intake for vitamin D that was not based on the level that provided maximal benefit but on the level that benefited the majority of the population. Based on that, a vitamin D level of 12 ng/ml was defined as a risk of deficiency, 12-20 ng/ml as a risk of inadequacy, and 20-50 ng/ml as adequate levels [36].

It has been generally accepted that individuals living between latitudes 40° North and 40° South are exposed to high and sufficient ultraviolet B (UVB) radiation. Hence, vitamin D production in those individuals is expected to be maintained at normal levels throughout the year [37]. Accordingly, those living outside this range were considered deficient in sufficient UVB exposure, especially in the wintertime [33]. However, the prevalence of vitamin D deficiency has been increasing in both low (within the tropics) and high (outside the tropics) latitudes and has become a global public health problem [38-40]. The long-held belief that geographical location is the fundamental determinant of vitamin D status was based on the assumption that exposure of the face and arms to UVB (290-370 nm) for 15-30 minutes between 11 am and 3 pm is quite sufficient to maintain normal vitamin D blood levels [35]. Therefore, the prevalence of vitamin D deficiency in sunny countries could reflect the significance of other factors affecting vitamin D synthesis in those areas, such as clothing habits, skin pigmentation, pollution, and genetic factors [33]. The complex relation between vitamin D synthesis in the skin and sunlight availability and actual exposure necessitates a better understanding of the impact of sunlight on vitamin D levels based on individual exposure and not only the availability of UVB radiation [41,42].

In this study, serum magnesium levels were significantly higher in the sunlight non-exposed groups when compared to the exposed groups. Still, all groups had blood magnesium levels within the normal range (1.8-2.3 mg/dl) [42]. The routine assessment of blood magnesium within the context of assessing vitamin D status is in some way overlooked. In fact, magnesium deficiency remains clearly unaddressed in vitamin D deficiency. This could be due to the difficulty in screening for chronic magnesium deficiency, as normal serum level may still be associated with moderate-to-severe magnesium deficiency [24]. Magnesium helps in the activation of vitamin D, thereby assisting the regulation of calcium and phosphate and, consequently, bone growth. Furthermore, magnesium is required by enzymes that metabolize vitamin D [22]. Similar to vitamin D, magnesium deficiency is associated with various disorders, including skeletal deformities [18]. It has been reported previously that serum vitamin D concentrations were lower in subjects with magnesium deficiency and may even remain lower for two weeks after parenteral magnesium therapy [43]. Additionally, adequate magnesium supplementation is recommended in the treatment of vitamin D deficiency [24]. The results so far suggested that the relatively lower blood vitamin D levels in all study subjects could not be linked to less sunlight exposure or magnesium deficiency. There was no evidence that exposure to sunlight could alter the blood ionized magnesium level. In fact, neither fluorescent lighting nor sunlight exposure could alter ionized calcium and magnesium levels in an experimental setting, while vitamin D levels were significantly greater in the same setting [44].

Further analysis of our data revealed more interesting findings. Vitamin D levels were significantly higher in Bahrainis exposed to sunlight when compared to their expatriate counterparts. Most of the expatriate workers enrolled in this study were from India, Bangladesh, and Pakistan. People from those countries normally have pigmented skin, which could explain their vitamin D deficiency despite exposure to sunlight. Supporting our findings, Nimitphong and Holick reported that the prevalence of vitamin D deficiency was about 70% or higher in South Asia. The authors concluded that skin pigmentation, aging, and the application of sunscreen were the major determinants of the altered vitamin D status. Religious, lifestyle, and nutritional differences were also correlated contributors to vitamin D deficiency [38]. Moreover, in an estimation of vitamin D insufficiency in Germany, it was reported that people with colored skin often suffer from vitamin D deficiency [45]. So, at least in part, this could explain, following exposure to sunlight, why expatriates had lower vitamin levels when compared to Bahraini subjects. However, in the non-exposed Bahraini and expatriate groups, vitamin D levels were completely the opposite. Also, vitamin D levels in the non-exposed expatriates were significantly higher than expatriate workers exposed to sunlight. A possible assumption is that exposure to sunlight may not be the main determinant of vitamin D status. In favor of our results, the high prevalence of vitamin D deficiency in Brazil has been reported previously despite the fact that the country has both the lines of equator and Capricorn running through it [33]. Although magnesium blood levels were significantly higher in expatriate workers exposed to sunlight when compared to their Bahraini counterparts, magnesium levels remained within the normal range in all the study groups. Ethnic-related differences in dietary magnesium intake have been shown to alter magnesium levels [31]. We concluded that, at least in our hands, regardless of ethnicity or sunlight exposure, magnesium deficiency could not be detected and could not be linked to vitamin D deficiency in the studied population. Nevertheless, skeletal or intracellular magnesium deficiency could persist even in the presence of normal blood levels [18].

Limitations

The sample size was a challenge, and we believe it should be increased in future studies to consolidate our findings. Other variables relevant to vitamin D and magnesium, such as their reference intake and the presence of chronic diseases, should also be studied in the same population.

## Conclusions

Vitamin D is well known for being essential to bone integrity as well as its involvement in different physiological processes. Our data support the necessity of establishing country- and ethnic-specific definitions and guidelines for vitamin D status and sunlight exposure recommendation. The assessment of magnesium status should be an integral part of the overall assessment of vitamin D deficiency. 
